# Research trends in the *Korean Journal of Women Health Nursing* from 2011 to 2021: a quantitative content analysis

**DOI:** 10.4069/kjwhn.2023.06.20.1

**Published:** 2023-06-30

**Authors:** Ju-Hee Nho, Sookkyoung Park

**Affiliations:** College of Nursing, Jeonbuk National University, Jeonju, Korea

**Keywords:** Education, Health, Nursing, Research, Women

## Abstract

**Purpose:**

Topic modeling is a text mining technique that extracts concepts from textual data and uncovers semantic structures and potential knowledge frameworks within context. This study aimed to identify major keywords and network structures for each major topic to discern research trends in women’s health nursing published in the *Korean Journal of Women Health Nursing* (KJWHN) using text network analysis and topic modeling.

**Methods:**

The study targeted papers with English abstracts among 373 articles published in KJWHN from January 2011 to December 2021. Text network analysis and topic modeling were employed, and the analysis consisted of five steps: (1) data collection, (2) word extraction and refinement, (3) extraction of keywords and creation of networks, (4) network centrality analysis and key topic selection, and (5) topic modeling.

**Results:**

Six major keywords, each corresponding to a topic, were extracted through topic modeling analysis: “gynecologic neoplasms,” “menopausal health,” “health behavior,” “infertility,” “women’s health in transition,” and “nursing education for women.”

**Conclusion:**

The latent topics from the target studies primarily focused on the health of women across all age groups. Research related to women’s health is evolving with changing times and warrants further progress in the future. Future research on women’s health nursing should explore various topics that reflect changes in social trends, and research methods should be diversified accordingly.

## Introduction

Understanding research trends in a field is essential for its development [1]. This is particularly true in the field of women’s health nursing, which encompasses the unique area of reproductive health, including pregnancy and childbirth. These issues are not only relevant to women, but also have significant implications for the future of humanity [2]. Recent years have seen an increase in the number of women participating in economic activities, leading to social phenomena such as delayed marriage, non-marriage, and declining birth rates [3,4]. To address the low birthrate problem faced by South Korea (hereafter Korea), a national and institutional approach is required. Moreover, given the rapid changes in health levels experienced by women during life stages such as pregnancy, childbirth, and menopause [5], it is crucial to develop evidence-based health policies specifically tailored to women. Consequently, it is important to evaluate how the focus and subject matter of research related to women’s health have evolved and to prepare for the way forward.

The quantitative analysis of large data sets is imperative for identifying key research concepts, trends, and expanding research areas [1]. Traditional literature analysis methods have been criticized for their inability to comprehensively identify central themes and major discussions [6]. In response, recent academic research has explored the use of big data analysis techniques, such as text network analysis and topic modeling, to identify research trends in specific fields [7]. Keyword network analysis is a technique that extracts significant words from text, identifies connections between them, and restructures them into a visual network [8]. Topic modeling, on the other hand, is an analytical method that uncovers latent keywords within text and examines the relationships and distributions of each topic [9]. By extracting concepts from textual data and identifying the semantic structure and potential knowledge structure within the context [10], topic modeling offers the advantage of considering multiple topics within a single document. This method is widely employed in management, policy, and industrial research [8] and has recently been applied in nursing.

Some studies have sought to identify research trends in women’s health by conducting network analyses of studies published in Korea. However, Jeon and colleagues’ study of manuscripts published in the Korean Journal of Women Health Nursing (KJWHN) up to 2018 [1] did not include centrality analysis, leading to limitations in identifying the influence of keywords. In addition, since Lee and Nho’s study [11] focused solely on middle-aged Korean women, it is challenging to analyze the trends in women’s health nursing research in Korea. Therefore, this study employed text network analysis and topic modeling to identify the keywords and network structure of recently published studies in KJWHN, with the aim of exploring the knowledge structure and determining research trends in women’s health nursing in Korea. Our specific objectives were: (1) to investigate the structure and characteristics of the created network, (2) to identify major topics through topic modeling of studies published in KJWHN, and (3) to evaluate the network of keywords by topic of research published in KJWHN.

## Methods

Ethics statement: This study was conducted after receiving an exemption from Jeonbuk National University (2022-04-013) as a secondary data analysis of published materials without exposing sensitive information.

### Study design

This study employed a descriptive research design to identify the main concepts and research topics in KJWHN, using quantitative content analysis of text networks and topic modeling.

### Research procedures

The study targeted manuscripts with English abstracts among the 373 papers published in KJWHN from January 2011 to December 2021. The research process involved: (1) data collection, (2) word extraction and refinement, (3) keyword extraction and network creation, (4) network centrality analysis and key topic selection, and (5) topic modeling analysis.

### Data collection

Data for this study were collected in February 2022 to identify research trends in KJWHN over the past decade, from January 1, 2011 to December 31, 2021. Full-text articles were accessed from the journal website (https://kjwhn.org/), and published papers with English abstracts were identified. Out of the 379 papers published during this period, 373 were analyzed, excluding six papers that did not have English abstracts available. The analyzed papers were organized by unique number, title, author, year of publication, abstract, and keywords using the MS Office Excel program (Microsoft, Redmond, WA, USA). Typographical errors and English spelling were checked using the spell-check function of the Excel program. Additionally, words such as “background,” “objectives,” “purpose,” “methods,” “results,” and “conclusion,” which are frequently used in standard abstracts, were removed.

### Word extraction and refinement

Our research team utilized NetMiner (version 4.3; Cyram, Seongnam, Korea) to conduct a semantic network analysis, extracting keywords from the titles, English abstracts, and keywords of articles. The keywords were derived from both the title and abstract, not solely from the author’s keywords. In order to extract and refine these keywords, the researchers repeatedly read and discussed words and abbreviations with the same meaning, refining them into a single word and unifying them with similar words. As a result, 56 similar words were identified. For instance, “breastfeeding” and “breast feeding,” which can differ in spacing, were unified as “breastfeeding.” Likewise, “self-efficacy” and “self efficacy” were unified as “self-efficacy.” When a combination of uppercase letters, lowercase letters, noun phrases, and abbreviations was used, a representative word was designated. For example, “quality of life,” “qol,” and “QOL” were designated as “QOL” to avoid redundancy.

Next, stop words such as pronouns and numbers were excluded using the automatic filtering function within the NetMiner program. General verbs and auxiliary verbs, such as “do,” “make,” “use,” “would,” “could,” and “should,” were excluded through discussion among researchers. Unnecessary nouns and other parts of speech, including “one,” “two,” “participants,” “subjects,” “level,” “group,” “data,” “research,” “test,” “year,” “design,” “day,” “example,” “effect,” “as,” “without,” “though,” and “all,” which were irrelevant for analyzing trends in women’s health nursing research, were also excluded. Additionally, words indicating logical relationships such as “only,” “before,” “toward,” “to,” and “with,” as well as adverbs and prepositions, were excluded. Consequently, a list of synonyms and negative words, which the researchers had discussed and agreed upon, was entered into the NetMiner dictionary. Among the words extracted from the 373 English abstracts, those with a frequency of 10 or more occurrences were selected as keywords. From this group, the top 25 words with the highest frequency of simple occurrences were chosen as keywords.

### Extraction of keywords and creation of networks

Keyword network analysis identifies various characteristics by extracting significant words from the text, determining the connections between them, and reorganizing them into a visual network [12]. A network was created based on the co-occurrence relationships between words, using the total frequency (weight) of word pair co-occurrences. The window size was set to three, and the link frequency threshold was set to two in order to extract all relationships that appeared more than once. The direction was set to nondirectional, allowing for the formation of a network between keywords regardless of the order in which they appeared. Additionally, the “remove self-loop” option was set to “yes” to exclude identical keyword relationships.

### Network centrality analysis and key topic selection

Our research team performed a centrality analysis to assess the impact of particular keywords on the entire network. This analysis focused on refined keywords and employed a mediation centrality approach to ascertain their positions within the network [13]. Furthermore, we conducted a word cloud analysis [14] to provide a visual representation of significant keywords at a glance. The word cloud was generated by considering documents with a term frequency-inverse document frequency (TF-IDF) of 0.1 or higher, specifically targeting refined keywords.

### Topic modeling

Topics were extracted using the latent Dirichlet allocation (LDA) topic modeling technique after text preprocessing [12]. LDA is the most widely employed document generation model in text mining analysis [9]. To obtain meaningful results through topic modeling, the number of topics is crucial and must be determined by the researcher [9]. Consequently, this study focused on 4 to 8 topics, and multiple analyses were conducted. Furthermore, after setting the alpha (α) value to 0.1, the beta (β) value to 0.01, and the number of sampling repetitions to 1,000, the number of topics was compared and analyzed.

## Results

### Word extraction and refinement

**Figure 1.**
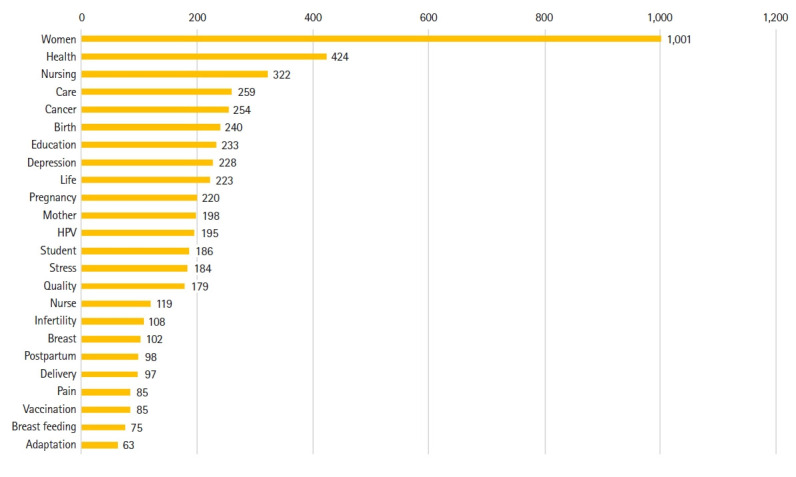
Top 25 keywords (term frequency). HPV: Human papillomavirus.

In total, 3,324 words were extracted and refined from the 373 article abstracts. The simple appearance frequencies of the extracted words were as follows, in descending order: “women” (1,001 instances), “health” (424 instances), “nursing” (322 instances), “care” (259 instances), “cancer” (254 instances), “birth” (240 instances), “education” (233 instances), “depression” (228 instances), “life” (223 instances), “pregnancy” (200 instances), “mother” (198 instances), “HPV” (195 instances), “stress” (186 instances), “quality” (184 instances), and “nurse” (179 instances) (Figure 1).

### Keyword network connection structure and centrality analysis

**Figure 2.**
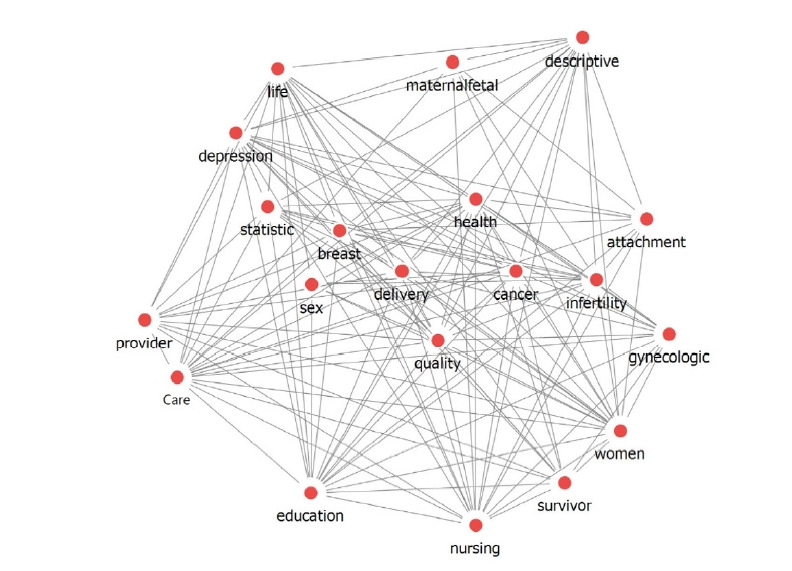
Network analysis by high-ranking co-occurrence between keywords.

**Figure 3.**
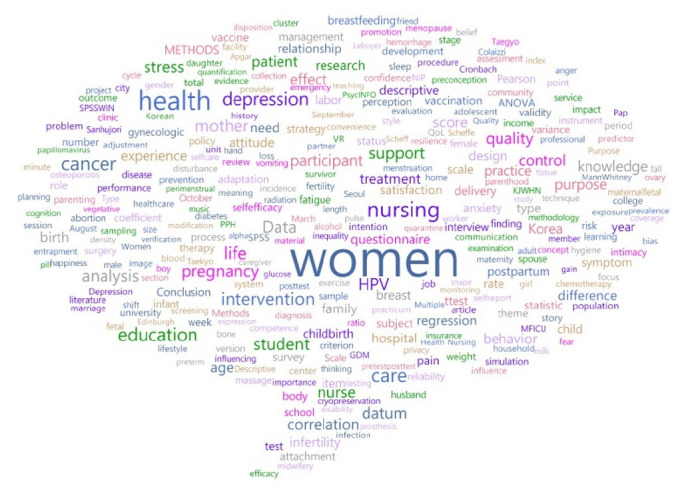
Word cloud (term frequency-inverse document frequency).

A network connection structure consisting of 3,425 nodes and 12,589 links was confirmed by examining the relationships between words. The density of the analyzed network was 0.015, the average connection degree was 9.054, and the average connection distance was 4.587 (Figure 2). Next, according to the TF-IDF analysis, the top keywords with high importance were “women” (198 instances), “health” (197 instances), “nursing” (189 instances), and “care” (178 instances). “education” (was 178 instances), and “life” (was 171 instances) (Table 1). Degree and betweenness centrality were analyzed. The keywords with high degree centrality were “women,” “health,” “care,” “nursing,” and “pregnancy,” and those with high betweenness centrality were “women,” “intervention,” “stress,” “experience,” and “nursing” (Table 1). A word cloud analysis presenting these high-importance keywords is shown in Figure 3.

### Topics and keywords identified through topic modeling

Six topics were identified and are presented in Table 2, along with their associated keywords and high allocation probabilities. Two researchers discussed and named the topics, taking into account the main keywords and connectivity for each topic extracted through topic modeling analysis. The six topics were named as follows: “gynecologic neoplasms,” “‘menopausal health,” “health behavior,” “infertility,” “women’s health in transition,” and “nursing education for women.” The proportions of each topic among the research papers were relatively even. Oncology-related research accounted for the highest proportion (33.3%), and education-related research exhibited the lowest proportion (10.7%) (Table 2). For the first topic (*gynecologic neoplasms*), the keywords were “permission,” “instrument,” “cm,” “tumor,” “modality,” “breast,” “survivor,” “education,” “mastectomy,” “indicator,” “chemotherapy,” “radiation,” “side effect,” “phase,” and “intrauterine.” The keywords that constituted the second topic (*menopausal health*) were “bone,” “obesity,” “mineral,” “height,” “menopause,” “dietary,” “cross,” “glucose,” “GDM,” “regularity,” “QOL,” “body composition,” “food,” “intake,” and “energy.” The keywords in the third topic (*health behavior*) were: “hygiene,” “drinking,” “smoking,” “exercise,” “diet,” “worker,” “hypertension,” “education,” “physical activities,” “water,” “incontinence,” “prevention,” “evaluation,” “women,” and “constipation.” The fourth topic (*infertility*) included the following keywords: “in vitro,” “IVF,” “failure,” “fact,” “laparoscopic,” “stigma,” “challenge,” “low,” “definition,” “format,” “choice,” “platform,” “welfare,” “decade,” and “primi.” The keywords found within the fifth topic were “recovery,” “translation,” “movement,” “caring,” “QOL,” “rest,” “nursing,” “orientation,” “behavioral,” “sexuality,” “functioning,” “reaction,” “medicine,” “midwifery,” and “flexibility,” and this topic was named *women’s health in transition*. Finally, keywords that constituted the sixth topic (*nursing education for women*) were “accuracy,” “education,” “educator,” “simulator,” “high fidelity,” “scenario,” “debriefing,” “childbearing,” “measuring,” “encouragement,” “context,” “professor,” and “facilitator.”

## Discussion

The degree centrality analysis, which measures influence through the number of connections to peripheral keywords, revealed the following terms: “women,” “health,” “care,” “nursing,” and “pregnancy.” This indicates that ongoing research is focused on improving women’s health, nursing, treatment management, and pregnancy-related health. Moreover, among the top 25 frequently appearing keywords, “exercise” and “attachment” disappeared from the ranking, while “menopause,” “body composition,” and “obesity” emerged as new entries. This suggests that health issues related to menopausal women have received increasing attention over the past decade. A previous analysis of papers published in KJWHN between 2013 and 2017 found that there were four times more studies on menopausal women than on elderly women [15]. Although our study’s timeframe overlapped with this prior analysis, our overall findings were consistent when including the subsequent 5 years (2017–2021). Since menopausal and midlife health significantly impacts health in later life, research on menopausal women’s health is essential [16]. Furthermore, given the prevalence of various physical symptoms and health problems among menopausal women, as well as the aging demographics of Korean women, sustained interest and research in this field are necessary.

“Obesity” was another top extracted keyword. Although a direct comparison is challenging due to the lack of text network analysis studies for women of all ages, our findings align with Lee and Noh’s [11] topic modeling analysis of health-related trends among middle-aged Korean women. Their study reported that obesity was the most frequently appearing keyword in the past decade [11]. Obesity rates continue to rise in Korea, and women are experiencing various health issues associated with it. Our findings suggest that obesity research appears to be active in Korea, and more studies may be needed to explore the relationships and impacts of women’s physical, mental, and social problems related to obesity.

Keywords with high betweenness centrality serve as crucial connectors, linking different groups of elements in the network [13]. These keywords should be considered when exploring related subjects. “Nursing” was present in four of the top 25 keywords ranked by frequency, and five keywords disappeared from the previous betweenness centrality ranking (“care,” “birth,” “life,” “mother,” and “attachment”). These were replaced by “risk factors,” “physical activity,” “satisfaction,” “lifestyle,” and “health promotion” as newly identified keywords. Similar findings were observed in the analysis of degree centrality and betweenness centrality. In other words, KJWHN’s main research topics have expanded to a wider spectrum of topics, covering not only pregnancy, childbirth, and family, but also various women’s health problems that are not related to maternity. This implies that research on lifestyle factors and health promotion has increased.

The topic that appeared most frequently in KJWHN during the selected period was gynecologic neoplasms, unlike what was reported in a previous analysis of KJWHN articles from 2008 to 2018, where “sexual health” was the most common research topic [1]. Considering our study period of 2011 to 2021, the number of women with cancer in Korea has increased from 102,357 in 2010 to 117,334 in 2020, at an average annual rate of 10.9% [17]. Accordingly, research on women with cancer has also increased, and this trend may be expected to continue in light of the increasing number of cancer survivors.

The next most frequently occurring topics were *menopausal health and health behavior*. Menopausal women have been reported to be relatively vulnerable and often lacking in s self-care [18]. As discussed earlier, the growing interest in middle-aged women’s health is expected to lead to continuing nursing studies on postmenopausal women’s health management. Given the importance of *health behavior* and the fact that research has been conducted not only on pregnancy, childbirth, and diseases, but also on women’s lifestyle improvement and health promotion behaviors, more studies on health promotion at midlife are also needed for the future.

The next extracted category, *infertility*, likely reflects the recent rise in infertility cases among Korean women [19]. Women experiencing infertility often face numerous physical and psychological challenges, which can impact their quality of life [20]. Given the ultra-low birth rate in Korea and its implications for infertility care [21], it is essential for future research to focus on preparing nurses to deliver improved care for women dealing with infertility.

In relation to the fifth extracted category, *women’s health in transition* women undergo various life transitions, such as pregnancy, childbirth, menopause, and aging, which affect their physical, social, and mental well-being [22]. Nurses must comprehend these transitional phases and identify strategic ways to improve women’s health. Consequently, future research may be needed on women’s transition processes, particularly focusing on health-promoting behaviors and adjustments during transitional periods.

The final extracted category was *nursing education for women*. Specifically, there has been a growing number of studies on this topic as various learning methods, such as simulation and virtual reality, are being employed to enhance the educational environment for women’s health [23]. This significant subject not only impacts women’s healthcare but also aligns with KJWHN’s aims and scope. As technological advances in learning will continue in the future, ongoing research on education in the field of women’s health nursing is essential.

This study had some limitations. The analysis only considered studies from the last decade with English abstracts, which may have influenced our findings. KJWHN publishes issue papers and statistical/methods papers in addition to original papers, necessitating careful interpretation. Another limitation is that some identified keywords were common concepts inherently related to the journal’s nature (e.g., women, health, nursing). Future analyses of research trends may consider excluding common keywords to focus on a more refined analysis and interpretation. Greater objectivity can be achieved in future research by repeating research methods with the totality of studies that have been published to date and enlisting experts to review the process of refining terms during network analysis. Despite these limitations, this study extracted potential topics based on text network and topic modeling analysis, classified research topics, and identified six major research topics from studies published in KJWHN. This is significant not only for presenting research trends in women’s health nursing, but also for considering research areas that warrant further attention in the future. By understanding the flow and characteristics of recent research, we were able to identify that women’s health-related research is changing to reflect social changes in Korea. Our findings will be meaningful in suggesting future directions for women’s health research.

## Figures and Tables

**Table 1.** Top 25 keywords (TF-IDF) and centrality analysis results for keywords

Rank	Keywords	TF-IDF	Keywords	Degree centrality	Keywords	Betweenness centrality
1	Women	198	Women	0.454	Women	0.109
2	Health	197	Health	0.342	Intervention	0.099
3	Nursing	189	Care	0.305	Stress	0.093
4	Care	178	Nursing	0.295	Experience	0.093
5	Education	178	Pregnancy	0.293	Nursing	0.089
6	Life	171	Education	0.236	Health	0.089
7	Pregnancy	168	Life	0.195	Risk factors	0.084
8	Cancer	162	Breastfeeding	0.186	Education	0.084
9	Depression	157	Family	0.185	Physical activity	0.082
10	Student	155	Anxiety	0.185	Satisfaction	0.054
11	Stress	147	Cancer	0.175	Pregnancy	0.053
12	Exercise	143	Postpartum	0.170	Lifestyle	0.050
13	Mother	139	Depression	0.166	Welfare	0.043
14	Birth	121	Stress	0.161	Cancer	0.038
15	Fatigue	121	Infertility	0.159	Depression	0.038
16	Postpartum	120	Menopause	0.158	Body composition	0.033
17	Quality	118	Mother	0.157	Postpartum	0.031
18	Breastfeeding	114	Birth	0.151	Health promotion	0.031
19	Intervention	112	Body Composition	0.148	Self-efficacy	0.021
20	Infertility	113	Child	0.110	Anxiety	0.017
21	Infant	110	Obesity	0.101	Fitness	0.014
22	Fertility	106	Welfare	0.086	Infertility	0.012
23	Breastfeeding	98	Intervention	0.086	Breastfeeding	0.011
24	Attachment	96	Self-efficacy	0.075	Child	0.010
25	Self-efficacy	92	Breast	0.069	Breast	0.010

TF-IDF: Term frequency-inverse document frequency.

**Table 2.** Results of topic modeling

Rank	Topic 1	Topic 2	Topic 3	Topic 4	Topic 5	Topic 6
	Gynecologic neoplasms	Menopausal health	Health behavior	Infertility	Women’s health in transition	Nursing education for women
%	33.3	15.3	15.1	12.8	12.8	10.7
1	Permission (.115)	Bone (.062)	Hygiene (.057)	Vitro (.055)	Recovery (.056)	Accuracy (.051)
2	Instrument (.069)	Obesity (.052)	Drinking (.044)	IVF (.037)	Translation (.040)	Education (.028)
3	Cm (.036)	Mineral (.044)	Smoking (.044)	Failure (.034)	Movement (.039)	Educator (.025)
4	Tumor (.030)	Height (.042)	Exercise (.042)	Fact (.033)	Caring (.037)	Simulator (.022)
5	Modality breast (.028)	Menopause (.040)	Diet (.041)	Laparoscopic (.029)	QOL (.037)	High fidelity (.017)
6	Survivor (.028)	Dietary (.035)	Worker (.034)	Stigma (.029)	Rest (.036)	Scenario (.013)
7	Education (.027)	Cross (.033)	Hypertension (.028)	Challenge (.027)	Nursing (.034)	Debriefing (.011)
8	Mastectomy (.027)	Glucose (.030)	Education (.027)	Low (.025)	Orientation (.033)	Childbearing (.009)
9	Indicator (.024)	GDM (.030)	Physical activities (.026)	Definition (.024)	Behavioral (.027)	Measuring (.007)
10	Chemotherapy (.021)	Regularity (.029)	Water (.019)	Format (.022)	Sexuality (.025)	Encouragement (.007)

GDM: Gestational diabetes mellitus: IVF, *in vitro* fertilization: QOL, quality of life.

## References

[1] Jun EM, Kang SW, Kang C (2021). A trend analysis of Korean Journal of Women Health Nursing using by topic model. J Korean Data Anal Soc.

[2] Yoon EK, Kim TW (2020). Research trends and prospects of medical anthropology: concepts and their intersection with history of medicine. Korean J Med Hist.

[3] Ma L (2016). Female labour force participation and second birth rates in South Korea. J Popul Res.

[4] Lee SY, Hwang MJ (2014). A trend analysis on non-married persons in accordance with educational attainment: comparison between Gangnam and Gangbuk in Seoul areas. J Soc Sci.

[5] Park JH, Bae SH, Jung YM (2020). Validity and reliability of the Korean version of the Menopause-Specific Quality of Life. J Korean Acad Nurs.

[6] Park JH, Chun M, Bae SH, Kim HJ (2021). Research trends on factors influencing the quality of life of cancer survivors: text network analysis and topic modeling approach. Asian Oncol Nurs.

[7] Kim K, Lee KS (2020). Identification of the knowledge structure of cancer survivors’ return to work and quality of life: a text network analysis. Int J Environ Res Public Health.

[8] Lee SS (2014). A content analysis of journal articles using the language network analysis methods. J Korean Soc Inf Manag.

[9] Song M (2017). Text mining.

[10] Baek YM (2017). Text-mining using R.

[11] Lee DY, Noh GO (2022). Research trends of middle-aged women’ health in Korea using topic modeling and text network analysis. J Converg Cult Techonol.

[12] Kim SM, Kim YJ (2020). Research trend analysis on living lab using text mining. J Digit Converg.

[13] Hwang SI, Shim JW (2020). Semantic network analysis of “smart city” in newspaper articles: from 2016 to 2019. J Digit Contents Soc.

[14] Kim JH, Mun HJ, Lee H (2021). A study on trend analysis in convergence research applying word cloud in Korea. JDC.

[15] Lee YJ, Kim SY, Kang SY, Kang YJ, Jin L, Jung HY (2018). Trend analysis of research articles published in the Korean Journal of Women Health Nursing from 2013 to 2017. Korean J Women Health Nurs.

[16] Jeong YW, Kang KI, Lee BJ (2020). A study on experiences of health problems and coping in middle-aged and elderly women in the community: focusing on focus group interview approach. J Korean Acad Community Health Nurs.

[17] National Cancer Information Center (2020). Cancer incidence [Internet]. https://www.cancer.go.kr/lay1/S1T639C640/contents.do.

[18] Chang HK (2012). Influencing factors on health related quality of life in middle age. Korean J Adult Nurs.

[19] Statistics Korea (2022). 2022 Birth statistics (confirmation) [Internet]. http://kostat.go.kr/portal/korea/kor_nw/1/2/index.board?bmode=read&aSeq=362574.

[20] Jung YJ, Kim HY (2017). Factors influencing infertility-related quality of life in women undergoing assisted reproductive techniques: focusing on depression and resilience. Korean J Women Health Nurs.

[21] Kim M (2021). National policies for infertility support and nursing strategies for patients affected by infertility in South Korea. Korean J Women Health Nurs.

[22] Meleis AI, Sawyer LM, Im EO, Hilfinger Messias DK, Schumacher K (2000). Experiencing transitions: an emerging middle-range theory. ANS Adv Nurs Sci.

[23] Chun N, Nho GO (2016). Quality improvement of clinical practice in nursing students: focused on delivery room clinical practice. J Korea Contents Assoc.

